# Monitoring of ventricular rate in atrial fibrillation patients through an app based on pulse-plethysmography

**DOI:** 10.1093/europace/euag161

**Published:** 2026-07-07

**Authors:** Anouk L de Ruiter, Ineke Baas-Arends, Marcelle D Smit, Robert G Tieleman

**Affiliations:** Dept of Cardiology, Martini Hospital Groningen, Van Swietenplein 1, Groningen 9728 NT, The Netherlands; Dept of Cardiology, Martini Hospital Groningen, Van Swietenplein 1, Groningen 9728 NT, The Netherlands; Dept of Cardiology, Martini Hospital Groningen, Van Swietenplein 1, Groningen 9728 NT, The Netherlands; Dept of Cardiology, Martini Hospital Groningen, Van Swietenplein 1, Groningen 9728 NT, The Netherlands

**Keywords:** Atrial Fibrillation, Pulse-plethysmography, Heart rate

## Introduction

Atrial fibrillation (AF) is the most prevalent cardiac arrhythmia in adults globally and is associated with high morbidity and mortality.^1,2^ Effective rate control is a key component in AF management, according to the European Society of Cardiology (ESC) guidelines, which recommend a resting heart rate below 110 bpm in most patients unless stricter control is indicated due to severe symptoms.^2,3^

During recent years, apps based on pulse-plethysmography (PPG) have been shown to be useful for the discrimination of AF from sinus rhythm.^4–6^ Furthermore, use of these apps has been advocated for the management of AF^7^ and guidance of rate control^8^ to reduce hospital visits and reduce the time needed to achieve adequate rate control.

While heart rate measurements using PPG have been validated for ventricular rates up to 110 bpm, accuracy at higher rates has not been established.^8^ From physical examination, it is known that at high rates during AF, a pulse deficit can be present.^9,10^ This could also potentially lead to underestimation of the rate by PPG-based technologies, which is particularly relevant when PPG is used to monitor the effect of rate control therapy in rapidly conducted AF.

## Methods

A single-centre prospective study was conducted at the Martini Hospital (Groningen, Netherlands) to evaluate the performance of a PPG app (Happitech) for heart rate monitoring in patients with AF. Heart rate measurements were performed on patients ≥18 years presenting with any type of AF in the emergency department, coronary care unit (CCU), or during consultations.

The measurements were performed using the app on an iPhone 7 Plus or an iPhone 13 Pro (Apple Inc., Cupertino, CA). Each patient was instructed to place their finger on the smartphone lens for 90 s to perform a measurement. At the end of a successful measurement, the app presented the rhythm (AF or no AF) and the average heart rate.

Simultaneously, the heart rate was recorded with ECG monitoring, either a 12-lead ECG or a continuous ECG monitor at the ward. The average heart rates as presented on the 12-lead ECG or bed-side monitor were compared with the heart rates measured by the app. In a subset of patients, additional measurements were performed using a second PPG-based app (*n* = 7, Fibricheck) and a single-lead ECG device (*n* = 12, KardiaMobile) for illustrative purposes. These devices were included to explore whether other digital tools showed similar patterns of accuracy or error at varying heart rates.

The study complies with the Declaration of Helsinki and approval was granted by the nWMO Advisory Committee (MEC 2025–045) of the Martini Hospital.

## Results

Sixty-one AF patients were studied (17 females, age 70 ± 13 years, CHA2DS2va 64% ≥ 2, treated with beta-blockers 53%, calcium-channel blockers 26%, digoxin 28%). A successful measurement with the Happitech app was obtained in 49 cases (80.3%). Among these, the app detected an irregular rhythm in 47 patients (95.9%).

Heart rate values obtained with the app showed a moderate positive correlation with ECG measurements (Pearson’s *r* = 0.653, *P* < 0.001). Regression analysis indicated that 42.7% of the variability in heart rate measured by the app could be explained by ECG heart rate (*R*^2^ = 0.427, *P* < 0.001).

In the subgroup of patients with an ECG heart rate above 110 bpm (*n* = 30), the accuracy of the PPG app declined markedly. In this group, the correlation between app and ECG heart rates was weak and non-significant (*r* = 0.099, *P* = 0.653). Furthermore, in 60.9% of these patients, the app underestimated heart rate by more than 20 bpm compared to ECG, and the difference ranged from 27 to 63 bpm. In 23.3% of high-rate cases, no successful app measurement was obtained at all.

**Figure 1:**
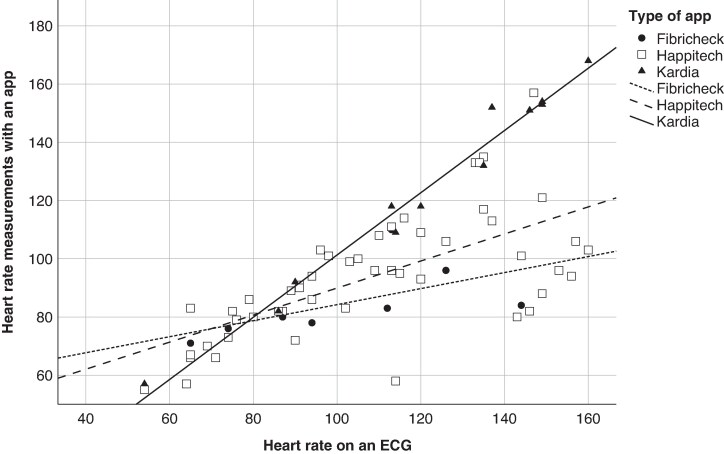
Scatter plot of heart rate measured by ECG plotted against heart rate measured with different apps. Round: Fibricheck, Square: Happitech, Triangle: Kardia.

Visual analysis of the data illustrates that the discrepancy between the app and ECG becomes more pronounced as heart rate increases (*Figure 1*). The Fibricheck app showed a similar pattern of reduced accuracy and underestimation at higher heart rates (*R*^2^ = 0344). In contrast, the Kardia 1-lead ECG device consistently provided accurate heart rate values, even at rates far above 110 bpm (*R*^2^ = 0976).

## Discussion

This study assessed the accuracy of a PPG app for heart rate measurement in AF patients by comparing it with ECG results. Overall, a moderate correlation was found (*r* = 0.653, *P* < 0.001), with ECG heart rate explaining 42.7% of the variation in app measurements (R^2^ = 0.427). However, in patients with heart rates >110 bpm, accuracy declined sharply (*r* = 0.099, *P* = 0.653), and 60.9% of these cases showed a deviation >20 bpm. In 23.3%, no valid measurement was obtained. The scatterplot illustrates increasing discrepancies at higher heart rates. Given that in a rate control strategy, the app is used to monitor whether heart rates reach the <110 bpm target, this limitation hampers its clinical utility. Similar inaccuracies were present in the exploratory observation with another PPG-based app (Fibricheck), while an ECG-based device (Kardia) remained accurate. The total number of patients is relatively low, which decreases statistical power and generalizability of the results. However, based on these results, additional research is required before PPG apps can be used clinically to monitor rate control in AF.

## Limitations

An important limitation of this study is that the heart rate measurements were not truly synchronous. An ECG captures only a 10-second snapshot, whereas the app requires a 90-second recording. Because heart rate in AF can fluctuate considerably, variation during the longer app measurement is inevitable and may affect the comparability of both methods. The same difficulty becomes apparent when examining continuous telemetry, where the degree of temporal variability makes it challenging to determine a single average heart rate that reliably reflects the underlying rhythm. However, this is also true for the Kardia measurements, which did show a strong correlation between the measured ECG/telemetry rates and the ECG-based app.

## Conclusion

Apps based on PPG show only moderate correlation with ECG in measuring heart rate in patients with AF, and its accuracy declines substantially at heart rates above 110 bpm. While PPG may be suitable for detecting the presence of AF, further research is required before PPG apps can be used clinically to monitor rate control in AF.

## Data Availability

The data underlying this article will be shared on reasonable request to the corresponding author.
